# SLAM Project - Long Term Ecological Study of the Impacts of Climate Change in the natural forest of Azores: I - the spiders from native forests of Terceira and Pico Islands (2012-2019)

**DOI:** 10.3897/BDJ.9.e69924

**Published:** 2021-09-01

**Authors:** Ricardo Costa, Paulo A. V. Borges

**Affiliations:** 1 cE3c – Centre for Ecology, Evolution and Environmental Changes / Azorean Biodiversity Group and Universidade dos Açores, Rua Capitão João d’Ávila, São Pedro, 9700-042, Angra do Heroismo, Azores, Portugal cE3c – Centre for Ecology, Evolution and Environmental Changes / Azorean Biodiversity Group and Universidade dos Açores, Rua Capitão João d’Ávila, São Pedro, 9700-042 Angra do Heroismo, Azores Portugal; 2 IUCN SSC Mid-Atlantic Islands Specialist Group,, Angra do Heroísmo, Azores, Portugal IUCN SSC Mid-Atlantic Islands Specialist Group, Angra do Heroísmo, Azores Portugal

**Keywords:** Arthropoda, Araneae, Macaronesia, Laurissilva forest, long-term sampling, SLAM traps

## Abstract

**Background:**

Long-term monitoring of invertebrate communities is needed to understand the impact of key biodiversity erosion drivers (e.g. habitat fragmentation and degradation, invasive species, pollution, climatic changes) on the biodiversity of these high diverse organisms.

The data we present are part of the long-term project SLAM (Long Term Ecological Study of the Impacts of Climate Change in the natural forest of Azores) that started in 2012, aiming to understand the impact of biodiversity erosion drivers on Azorean native forests (Azores, Macaronesia, Portugal). In this contribution, the design of the project, its objectives and the first available data for the spider fauna of two Islands (Pico and Terceira) are described.

Passive flight interception SLAM traps (Sea, Land and Air Malaise traps) were used to sample native forest plots in several Azorean islands, with one trap being set up at each plot and samples taken every three months following the seasons.

The key objectives of the SLAM project are: 1) collect long-term ecological data to evaluate species distributions and abundance at multiple spatial and temporal scales, responding to the Wallacean and Prestonian shortfalls, 2) identify biodiversity erosion drivers impacting oceanic indigenous assemblages under global change for conservation management purpose, 3) use species distribution and abundance data in model-based studies of environmental change in different islands, 4) contribute to clarifying the potential occurrence of an "insect decline" in Azores and identifying the spatial and temporal invasion patterns of exotic arthropod species, 5) contribute with temporal data to re-assess the Red-list status of Azorean endemic arthropods and 6) perform studies about the relationship between diversity (taxonomic, functional and phylogenetic) and ecosystem function.

**New information:**

The project SLAM (Long Term Ecological Study of the Impacts of Climate Change in the natural forest of Azores) is described in detail.

Seasonal distribution and abundance data of Azorean spiders, based on a long-term study undertaken between 2012 and 2019 in two Azorean Islands (Terceira and Pico), is presented. A total of 14979 specimens were collected, of which 6430 (43%) were adults. Despite the uncertainty of juvenile identification, juveniles are also included in the data presented in this paper, since the low diversity allows a relatively precise identification of this life-stage in Azores.

A total of 57 species, belonging to 50 genera and 17 families, were recorded from the area, which constitutes baseline information of spiders from the studied sites for future long-term comparisons. Linyphiidae were the richest and most abundant family, with 19 (33%) species and 5973 (40%) specimens. The ten most abundant species are composed mostly of endemic or native non-endemic species and only one exotic species (*Tenuiphantestenuis* (Blackwall, 1852)). Those ten most abundant species include 84% of all sampled specimens and are clearly the dominant species in the Azorean native forests. *Textrixcaudata* L. Koch, 1872 was firstly reported from Terceira and Pico Islands, *Araneusangulatus* Clerck, 1757 was firstly reported from Terceira Island, *Nerieneclathrata* (Sundevall, 1830) and *Macaroerisdiligens* (Blackwall, 1867) were firstly reported from Pico Island.

This publication contributes not only to a better knowledge of the arachnofauna present in native forests of Terceira and Pico, but also to understand the patterns of abundance and diversity of spider species, both seasonally and between years.

## Introduction

In the past decades, the Azorean terrestrial arthropod fauna (focusing on most arthropod groups, but excluding Acari, Collembola Diptera and Hymenoptera) has been extensively surveyed by a wide range of different studies, from community inventories on native habitats that started in the 1990s (Borges et al. 2005, Ribeiro et al. 2005, Borges et al. 2006), to those in areas differing in anthropogenic disturbance (Cardoso et al. 2009, Florencio et al. 2015, Marcelino et al. 2021). These studies contributed greatly, not only for finding new species and records for the Archipelago (e.g. Borges and Wunderlich 2008, Crespo et al. 2013, Crespo et al. 2014, Borges et al. 2017a) contributing to solving the Linnean shortfall, but also to solve other emergent biodiversity shortfalls, namely the Wallacean shortfall, knowing better the distribution of species (Borges et al. 2016) and the Prestonian shortfall, improving the knowledge on species relative abundances (Gaston et al. 2006, Rigal et al. 2013, Matthews et al. 2014). More importantly, all these new data allowed the recent red-listing of all Azorean endemic arthropods (see, for example, Borges et al. 2018a and http://www.maiisg.com/species/?archi=10&class=1).

In 2012, a long-term project to monitor the distribution and abundance of arthropods in native forests was set up in the Azores named as SLAM - "Long Term Ecological Study of the Impacts of Climate Change in the natural forest of Azores" (Borges et al. 2017b, Matthews et al. 2018, Borges et al. 2020). In this contribution, we describe this project and the first taxon for which data are readily available for the majority of the samples, i.e. spiders (Araneae).

Spiders are one of the most and better-studied arthropod groups of the Azores and make up an important part of the arthropod communities in these islands (Borges and Wunderlich 2008). Up to this date, a total of 131 species on 26 families are known to occur on the Azores, from which only 25 are endemic (Borges and Wunderlich 2008, Borges et al. 2010, Malumbres-Olarte et al. 2019). Although most species are introduced in the Archipelago, there is also a high percentage of endemics in the group when compared to other species diverse taxa, with endemic species comprising more than 60% of all indigenous spiders (Borges and Wunderlich 2008). In the past decades, many studies have aimed to understand diversity, abundance and phylogeographic patterns of this group, in both native and anthropogenic habitats, as well as their ecology and habitat affinities (Borges and Brown 2001, Borges and Brown 2004, Borges and Wunderlich 2008, Florencio et al. 2013, Florencio et al. 2015, Parmakelis et al. 2015, Rigal et al. 2017). Others have also focused on this group for inter-archipelago comparisons, trying to understand the reason behind the present patterns (Cardoso et al. 2010, Boieiro et al. 2018). Further works have also tried to detect which areas are of particular conservation interest in the Azores (Borges et al. 2005, Gaspar et al. 2011). The main threats to the Azorean spider fauna include the colonisation and spread of exotic species and habitat degradation (Borges and Wunderlich 2008, Cardoso et al. 2010), which may have already caused the extinction of many endemic spider species in the past (Borges and Wunderlich 2008, Cardoso et al. 2010, Boieiro et al. 2018, Crespo et al. 2021). The impact of climatic changes should also be considered as one of the main drivers of biodiversity erosion for this taxon in the Azores (Ferreira et al. 2016). Although most endemic species occur in most islands, others are restricted to one or two and also tend to be habitat specialists (Borges and Wunderlich 2008, Crespo et al. 2013, Crespo et al. 2014). However, the lack of population and demographic information is still a problem in making a proper assessment of the conservation status of many species (Borges and Wunderlich 2008).

Due to the need to better distinguish whether temporal trends on island environments are natural or are related to human activities (Magurran et al. 2010, Nogué et al. 2017), long-term monitoring schemes are extremely necessary (Magurran et al. 2010, Borges et al. 2018). The information they provide helps to better determine the conservation status of endemic species and to understand if the ecological and functional patterns observed vary or not amongst different habitats, islands and archipelagos (Rigal et al. 2017, Borges et al. 2018). Given the high abundance and diversity of endemic spiders in forest habitats, information about their population trends is particularly relevant to monitor the state of these environments (Borges and Wunderlich 2008, Cardoso et al. 2010, Borges et al. 2018), especially considering their sensitivity to changes in the habitat and abundance of prey (Pearce and Venier 2006, Cardoso et al. 2010).

In this contribution, we present the detailed distribution and abundance of Azorean spiders sampled during eight years (2012-2019) in two Islands (Pico and Terceira) within the project "SLAM - Long Term Ecological Study of the Impacts of Climate Change in the natural forest of Azores" that aims to understand the impact of biodiversity erosion drivers on Azorean native forests through time (Borges et al. 2017b, Matthews et al. 2018, Borges et al. 2020). In this way, we are contributing not only to a better knowledge of the arachnofauna present in the native forests of Pico and Terceira, but also to understand the patterns of spider species abundance and diversity, both seasonally and between years.

## General description

### Purpose

This publication is the first of a series that will explore time-series data in Azores native habitats, starting with the spider fauna in two Islands (Pico and Terceira). The data we present are part of the long-term project SLAM (Long Term Ecological Study of the Impacts of Climate Change in the natural forest of Azores) that started in 2012 aiming to understand the impact of biodiversity erosion drivers on Azorean native forests (Azores, Macaronesia, Portugal). Passive flight interception SLAM traps (Sea, Land and Air Malaise trap) were used to sample native forest plots in several Azorean islands, with one trap being set up at each plot.

With the current framework, we expect to accomplish the following objectives in the next years:

1) collect long-term ecological data to evaluate species distributions and abundance at multiple spatial and temporal scales, responding to the Wallacean and Prestonian shortfalls (Cardoso et al. 2011a);

2) identify biodiversity erosion drivers impacting oceanic indigenous assemblages under global change for conservation management purposes;

3) islands are especially good places to investigate species-environment relationships and we aim to use species distribution and abundance data in model-based studies of environmental change in different islands;

4) contribute to clarify the potential occurrence of an "insect decline" in Azores and identify the spatial and temporal invasion patterns of exotic arthropod species;

5) since long-term temporal data are mostly absent in invertebrates red listing (Cardoso et al. 2011b), we hope to contribute with temporal data to re-assess the Red-list status of Azorean endemic arthropods;

6) perform studies about the relationship between diversity (taxonomic, functional and phylogenetic) and ecosystem function.

### Additional information

The traps in Terceira Island have been operating since 2012 within the Project NETBIOME ISLANDBIODIV. In the other Islands (Flores, Faial, Pico, Graciosa, S. Miguel and S. Maria), the study started in August-September 2013. Since 2020, this project is being financed within the project LIFE-BEETLES for samples in the Islands of Flores, Pico and Terceira.

Since the start of the project, the Azorean Government has been supporting this project and Nature Parks rangers are giving support in collecting the samples every three months in all the abovementioned Islands with exception of Terceira Island, in which we are performing the monitoring.

## Project description

### Title

SLAM - Long Term Ecological Study of the Impacts of Climate Change in the natural forest of Azores

### Personnel

The project was conceived and led by Paulo A.V. Borges.

Fieldwork: (Terceira Island) - Alejandra Ros-Prieto, Fernando Pereira, Lucas Lamelas-López, Paulo A. V. Borges, Rui Carvalho, Rui Nunes; (Pico Island) - Paulo Freitas.

Parataxonomists: Adal Humberto Díaz Raya, Adrian Fernandez Marinez, Alba Arteaga, Alejandra Ros Prieto, Castore De Salvador, David Rodilla Rivas, Daniel Ehrhart, Elisa Tarantino, Gea Ghisolfi, Helena Marugán Páramo, Joel Martin Ay, Jonne Bonnet, Jose Vicente Pérez Santa Rita, Juan Ignacio Pitarch Peréz, Juan Manuel Taboada Alvarez, Laura Cáceres Sabater, Laura Gallardo, Magí Ramon Martorell, Maria Simitakou, Marija Tomašić, Marta Calera Sierra, Merili Martverk, Óscar García Contreras, Oscar Gomez-Novillo, Percy de Laminne de Bex, Reinier Vries, Riccardo Negroni, Ruben Murillo Garcia, Rui Carvalho, Rui Nunes, Sébastien Lhoumeau, Sergio Fernandez, Sophie Wallon and William Razey.

Taxonomists: Paulo A. V. Borges and Luís Carlos Crespo.

Voucher specimen management was mainly undertaken by Alejandra Ros Prieto and Paulo A. V. Borges.

### Study area description

**Figure 1.**
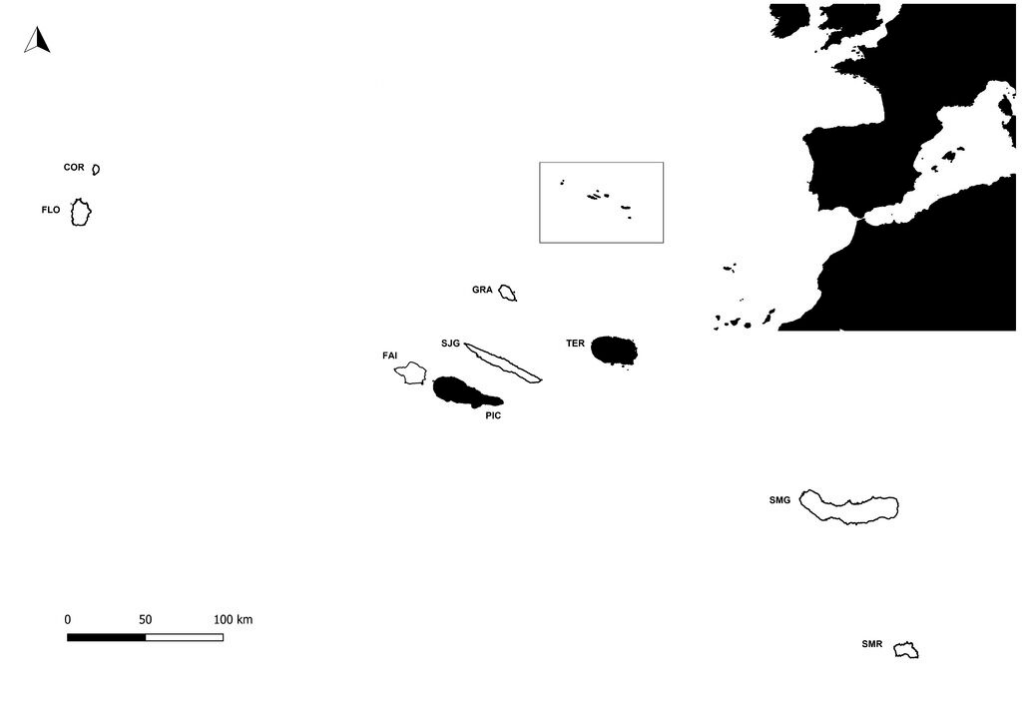
Map of the Azores Archipelago location in mid-Atlantic with the two studied Islands in black. COR - Corvo; FLO - Flores; FAI - Faial; PIC - Pico; SJG - São Jorge; GRA - Graciosa; TER - Terceira; SMG - São Miguel; SMR - Santa Maria.

During this project, several Islands (Flores, Faial, Pico, Graciosa, Terceira, S. Miguel and S. Maria) were surveyed in Azores (38°43′49″N, 27°19′10″W, Fig. 1), an Archipelago isolated in the mid-Atlantic Ocean, comprising nine volcanic Islands spread over 500 km in a WNW–ESE direction. Terceira and Pico in black in Fig. 1 were selected for the current manuscript.

**Figure 2.**
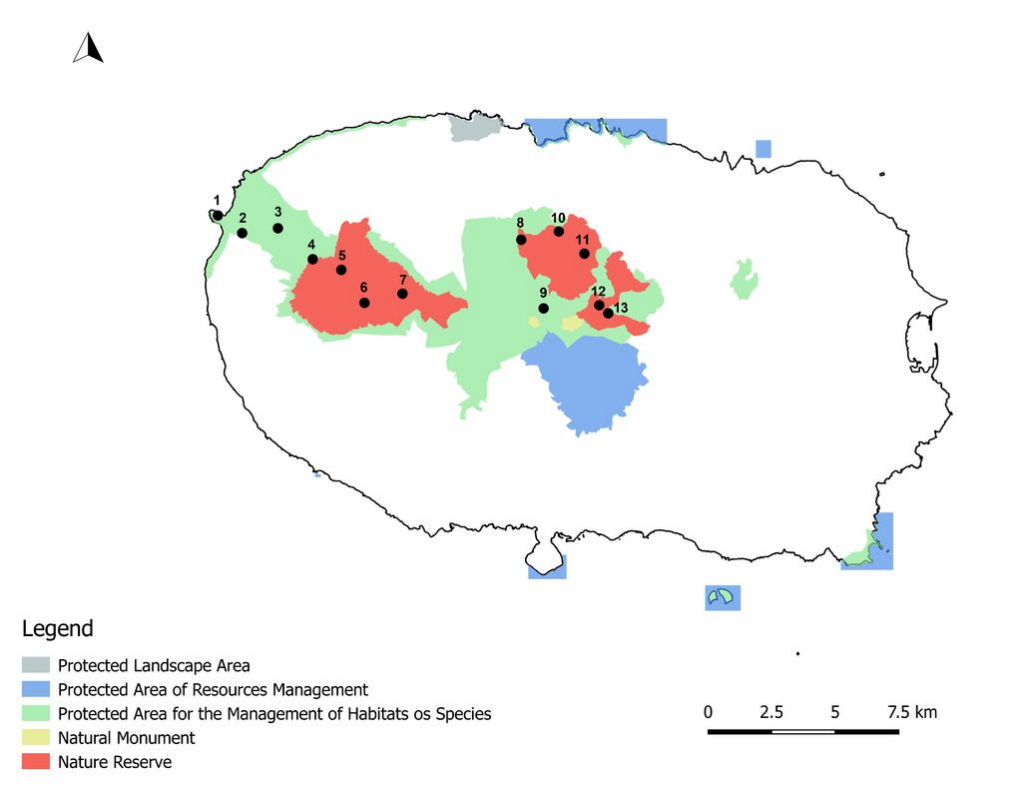
Sites in Terceira Island. 1. TER_0m; 2 -TER_200M; 3 - TER_400M; 4 - TER-NFSB-TE48; 5 - TER-NFSB-TE49; 6 - TER-NFSB-T164; 7 - TER-NFSB-T-07; 8 - TER-NFBF-T-02; 9 - TER-NFPG-T-33; 10 - TER-NFBF-T-01; 11 - TER-NFBF-TP41; 12 - TER-NFTB-T-15; 13 - TER-NFTB-T-18_Original.

**Figure 3.**
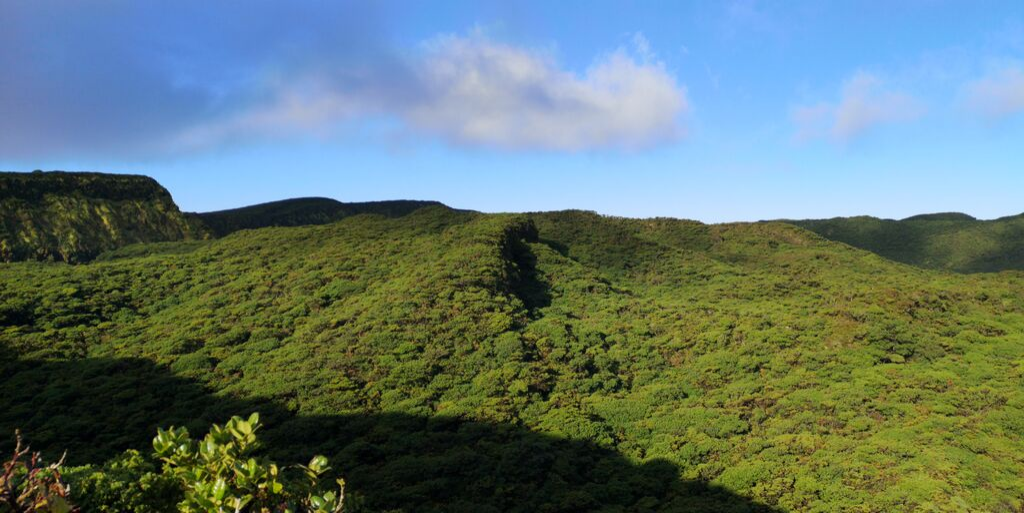
Caldeira St. Barbara, the most pristine area in Azores in which the site TER-NFSB-T164 is located (Credit: Paulo A. V. Borges).

Terceira is a roundish (402 km^2^) island formed by four main volcanic complexes (Serra de Santa Bárbara, Serra do Morião, Pico Alto and Serra do Cume), protected areas being located mostly in the Serra de Santa Bárbara in the western part of the Island and Pico Alto in the central part of the Island. The highest point (Serra de S. Bárbara, 1023 m) is simultaneously the most pristine area in the Azores (Gaspar et al. 2010) (Fig. 2; Fig. 3). The estimated geological age is around 0.4 Ma. (Ávila et al. 2016).

**Figure 4.**
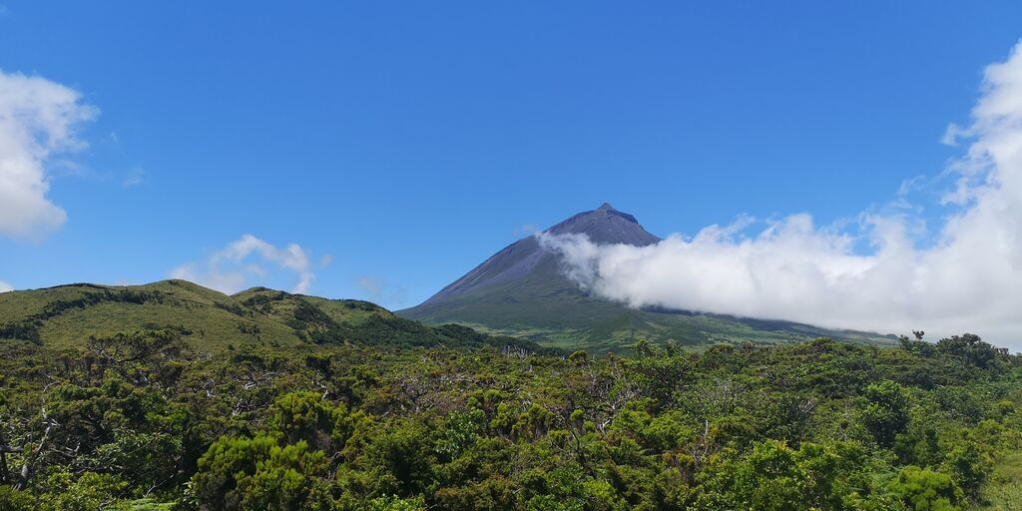
Pico Mountain, dominated by a strato-volcano (Pico Mountain) of 2351 m height (Credit: Paulo A. V. Borges).

**Figure 5.**
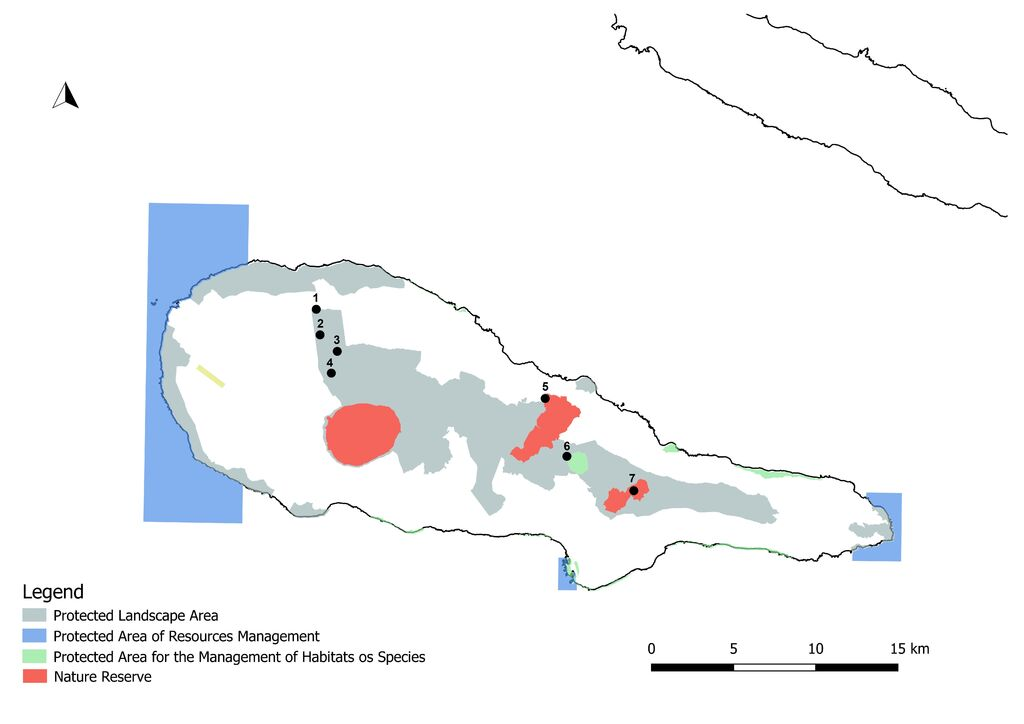
Sites in Pico island. 1 - PIC_ML_200; 2 - PIC_ML_400; 3 - PIC_ML_600; 4 - PIC_ML_800; 5 - PIC-NFMP-T-03; 6 - PIC-NFLC-T-02; 7 - PIC-NFCA-T-09.

Pico Island (436 km^2^) is the most recent of all the Azorean Islands with an estimated age of 0.19 Ma (Ávila et al. 2016) (Fig. 4; Fig. 5). The Island is dominated by a strato-volcano (Pico Mountain) with 2351 m elevation (Fig. 4). The Island is covered by old and more recent lava flows with plenty of lava tubes and volcanic pits.

The climate in the Azores is considerably influenced by the surrounding ocean and is characterised by a mild climate, with small fluctuations in temperature, large amounts of precipitation and high air humidity.

**Figure 6.**
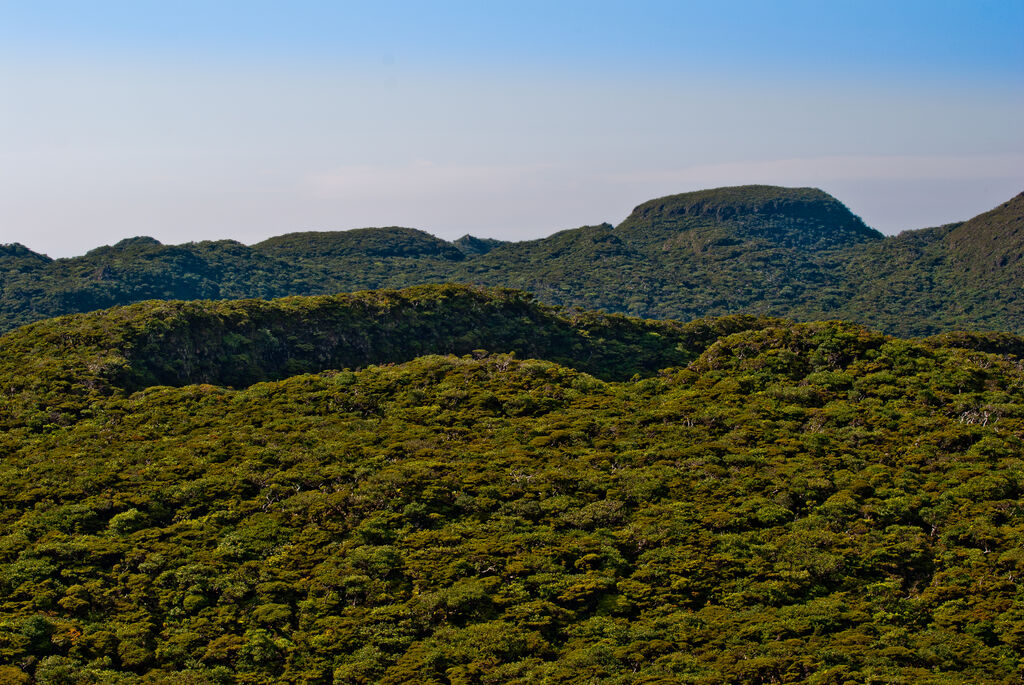
Native *Juniperus* woodlands at high elevation in Terceira Island (Credit: Paulo A. V. Borges).

**Figure 7.**
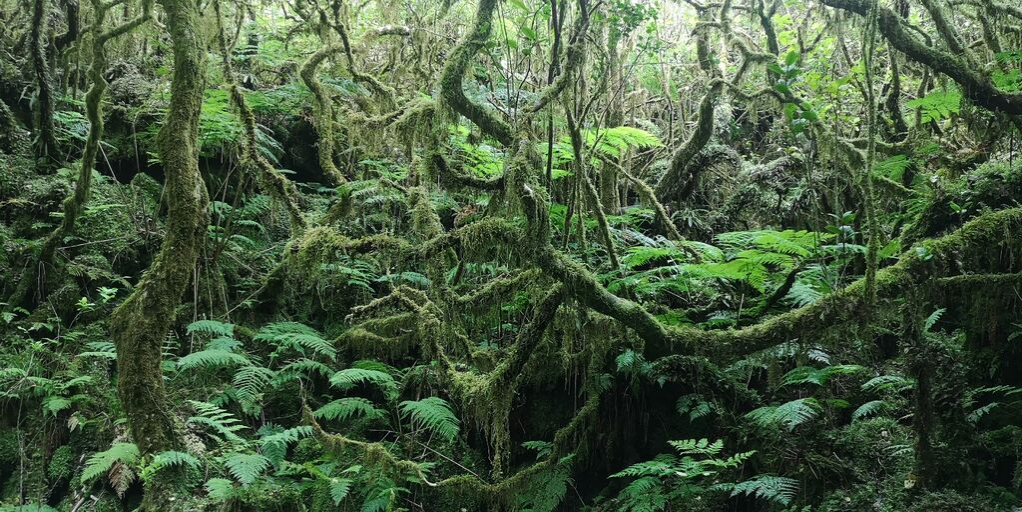
Dense cover of mosses and ferns in the hyper-humid Azorean native forests, in this case a Ilexperadosubsp.azorica forest in Mistério da Parinha at Pico Island (2020) (Credit: Paulo A. V. Borges).

Native vegetation is now restricted to high elevations with only about 5% of the original habitats still persisting and being protected, based on the IUCN criteria for protected areas (Gaspar et al. 2011). Small pockets of *Erica*-*Morella* woodland and *Picconia*-*Morella* forests are still persisting in the lowlands, up to 300 m a.s.l. in some Islands (Elias et al. 2016). However, between 600 m and 1000 a.s.l., *Juniperus*-*Ilex* forests and *Juniperus* woodlands are the current main native vegetation (Elias et al. 2016) (Fig. 6). Most of the extinct vegetation belongs to the Azorean Laurel forests, dominated by *Laurusazorica* that have probably covered more than two-thirds of the Islands, from 300 m to 600 a.s.l. (Elias et al. 2016). These forests are very dense and hyper-humid with a dense cover of bryophytes at all substrata and a dense cover of ferns in the soil (Fig. 7).

### Design description

The sampling referred to in this project has been performed in seven Azorean Islands (excluding Corvo and S. Jorge). The year in which the project started on each Island varied as well as the sampling frequency. Each sampling location was visited, in general, four times per year around the 15th March (winter sample), 15th June (spring sample), 15th September (summer sample) and 15th December (autumn sample). However, in some Islands (e.g. S. Maria and Graciosa) and sites (e.g. TER-NFTB-T-18_Original in Terceira), samples were obtained every month for some years. The specimens collected were taken to the laboratory for identification and preservation and the resulting vouchers were deposited at the Dalberto Teixeira Pombo Insect Collection of the University of the Azores.

### Funding

- FCT-NETBIOME –ISLANDBIODIV grant 0003/2011 (between 2012 and 2015) with a funding of around 60 k euros.
- EU ERASMUS +Training Grants to Ruben Murillo Garcia, Laura Gallardo (2014); Adal Humberto Díaz Raya, David Rodilla, Laura Cáceres Sabater, Óscar García Contrera, William Razey (2015); Alejandra Ros Prieto, Daniel Ehrhart, Helena Marugán Páramo, Maria Simitakou (2016); Juan Manuel Taboada Alvarez, Merili Martverk (2017); Elisa Tarantino, Marta Calera Sierra, Oscar Gomez-Novillo, Reinier Vries (2018); Adrian Fernandez Marinez, Castore De Salvador, Gea Ghisolfi, Joel Martin Aye; Jonne Bonnet, Riccardo Negroni (2019); Magí Ramon Martorell, Sébastien Lhoumeau (2021), with a total funding so far of around 70 k euros.
- EU EURODYSSÉE - Marija Tomašić (2014), Percy de Laminne de Bex, Juan Ignacio Pitarch Peréz (2015); Jose Vicente Pérez Santa Rita (2017); Alba Arteaga (2018), with a total funding so far of around 30 k euros.
- ESTAGIAR L Azores Government - Sophie Wallon (2014), with a funding of 12 k euros.
- ESTAGIAR T Azores Government - Alejandra Ros Prieto (2017), with a funding of 12 k euros.
- Portuguese National Funds, through FCT – Fundação para a Ciência e a Tecnologia, within the project UID/BIA/00329/2013-2020, with a funding of 9 k euros.
- Direcção Regional do Ambiente - PRIBES (LIFE17 IPE/PT/000010) (2019), with a funding of 6 k euros.
- Direcção Regional do Ambiente – LIFE-BETTLES (LIFE18 NAT_PT_000864) (2020), with a funding of 138 k euros until 2024.
- AZORESBIOPORTAL –PORBIOTA (ACORES-01-0145-FEDER-000072) (2019), with a funding of 9 k euros.

## Sampling methods

### Study extent

A total of twenty plots were sampled in two of the Islands from the Archipelago, thirteen in Terceira and seven in Pico (Table 1). The areas, where these plots were set, constitute some of the most well-preserved wet forests in these Islands, having only limited human disturbance (Borges et al. 2017b). In Terceira, ten of the plots (those with code TER-NF..) and three in Pico (those with code PIC-NF..) were originally set up within the project FCT-NETBIOME –ISLANDBIODIV (see Cicconardi et al. 2017, Malumbres-Olarte et al. 2019) and are dominated by endemic vegetation, such as *Juniperusbrevifolia*, *Ericaazorica*, *Laurusazorica* and Ilexperadosubsp.azorica (see Borges et al. 2017b for more details). In Pico, the plots at lower elevation (0-400 m a.s.l.) are dominated by *Ericaazorica* and *Morellafaya*, but with some presence of *Pittosporumundulatum*. At higher elevations (600-1000 m a.s.l.), the dominant vegetation is composed of *Laurusazorica*, *Juniperusbrevifolia* and Ilexperadosubsp.azorica.

### Sampling description

**Figure 8.**
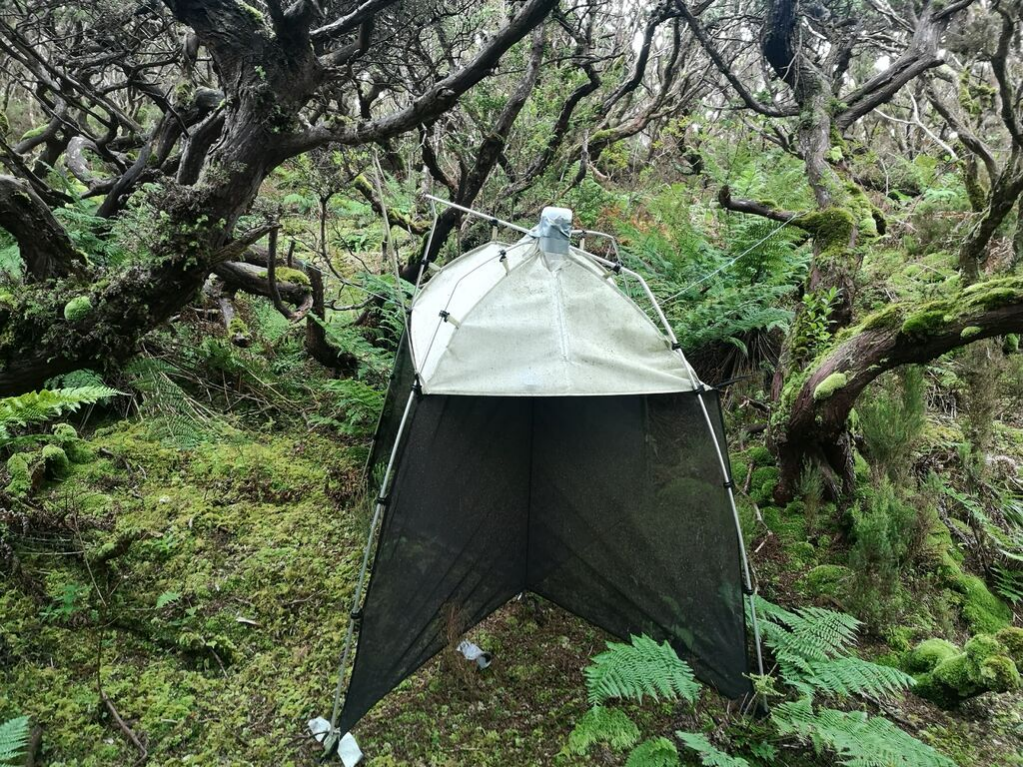
SLAM trap (Sea, Land and Air Malaise trap) in operation in a native forest from Terceira Island (Credit: Paulo A. V. Borges).

Passive flight interception SLAM traps (Sea, Land and Air Malaise trap) (Fig. 8) were used to sample the plots in both Islands, with one trap being set up at each plot, each one being 110 x 110 x 110 cm. In this type of trap, the trapped arthropods crawl up the mesh and then fall inside the sampling recipient (Borges et al. 2017b). Each one is filled with propylene glycol (pure 1,2-propanediol) to kill the captured arthropods and conserve the sample between collections, enabling also the preservation of DNA for future genetic analysis. Although this protocol was developed to sample flying arthropods, by working as an extension of the tree, non-flying species such as spiders, can also crawl into the trap (Borges et al. 2017b), enhancing the range of groups that can be sampled by this technique. Due to this, previous studies have used these traps to analyse diversity and abundance changes in the arthropod communities in Azores pristine forest sites (Matthews et al. 2018, Borges et al. 2020). The trap samples were collected every three months between September 2013 and December 2018 in Pico and between June 2012 and December 2019 in Terceira. A monthly collection was also performed in Terra-Brava T18 on Terceira, which happened between January 2014 and June 2016.

### Quality control

All sampled individuals were first sorted by trained paratoxonomists (see list above). All specimens were allocated to a taxonomic species by Paulo A. V. Borges. Despite the uncertainty of juvenile identification, juveniles are also included in the data presented in this paper, since the low diversity allowed a relatively precise identification of this life-stage in Azores.

For taxa for which it was not possible to assign a taxonomic name, a morphospecies code was created and voucher specimen(s) were sent to another taxonomic expert (Luís Crespo). The taxonomy follows the World Spider Catalogue (World-Spider-Catalog 2021).

### Step description

At the laboratory, specimen sorting and arthropod identification followed standard procedures. A combination of morphological and anatomical characters and reproductive structures was used for species identification. A reference collection was made for all collected specimens by assigning them a morphospecies code number and depositing them at the Dalberto Teixeira Pombo Insect Collection, University of Azores.

## Geographic coverage

### Description

Pico and Terceira Islands, the Azores, Macaronesia, Portugal

### Coordinates

38.372 and 38.835 Latitude; -28.592 and -26.993 Longitude.

## Taxonomic coverage

### Description

Araneae (Arthropoda, Arachnida)

### Taxa included

**Table:** 

Rank	Scientific Name	Common Name
order	Araneae	Spiders

## Traits coverage

Functional trait data including detailed morphometric measurements for most of the studied species can be accessed in the publication Macías-Hernández et al. 2020.

## Temporal coverage

### Notes

4 June 2012 to 13 January 2020 for Terceira Island and 2 September 2013 to 17 December 2018 for Pico Island.

## Collection data

### Collection name

Dalberto Teixeira Pombo insect collection at the University of Azores

### Collection identifier

DTP

### Specimen preservation method

All specimens were preserved in 96% ethanol.

### Curatorial unit

Dalberto Teixeira Pombo insect collection at the University of the Azores (Curator: Paulo A. V. Borges)

## Usage licence

### Usage licence

Creative Commons Public Domain Waiver (CC-Zero)

## Data resources

### Data package title

Long-term monitoring of Azorean forest spiders

### Resource link

https://www.gbif.org/dataset/13745243-620e-4c04-9178-773e4bfc2072

### Alternative identifiers

http://ipt.gbif.pt/ipt/archive.do?r=lter_slam_azores_spiders

### Number of data sets

1

### Data set 1.

#### Data set name

Long-term monitoring of Azorean forest spiders

#### Data format

Darwin Core Archive

#### Number of columns

56

#### Download URL

http://ipt.gbif.pt/ipt/resource?r=lter_slam_azores_spiders

#### Data format version

version 1.4

#### Description

The following data table includes all the records for which a taxonomic identification of the species was possible. The dataset submitted to GBIF (Global Biodiversity Information Facility) is structured as a sample event dataset, with two tables: event (as core) and occurrences. The data in this sampling event resource have been published as a Darwin Core Archive (DwCA), which is a standardied format for sharing biodiversity data as a set of one or more data tables. The core data file contains 495 records (eventID) and the occurrences file 3025 records (occurrenceID). This IPT (integrated publishing toolkit) archives the data and thus serves as the data repository. The data and resource metadata are available for download from Borges and Costa 2021.

**Data set 1.** 

Column label	Column description
Table of Sampling Events	Table with sampling events data (beginning of table).
id	Unique identification code for sampling event data.
eventID	Identifier of the events, unique for the dataset.
samplingProtocol	The sampling protocol used to capture the species.
sampleSizeValue	The numeric amount of time spent in each sampling.
sampleSizeUnit	The unit of the sample size value.
eventDate	Date or date range the record was collected.
year	Year of the event.
verbatimEventDate	The verbatim original representation of the date and time information for an Event. In this case, we use the season and year.
habitat	The habitat of the sample.
locationID	Identifier of the location.
islandGroup	Name of archipelago.
island	Name of the island.
country	Country of the sampling site.
countryCode	ISO code of the country of the sampling site.
stateProvince	Name of the region of the sampling site.
municipality	Municipality of the sampling site.
locality	Name of the locality.
minimumElevationInMetres	The lower limit of the range of elevation (altitude, usually above sea level), in metres.
locationRemarks	Details on the locality site.
decimalLatitude	Approximate centre point decimal latitude of the field site in GPS coordinates.
decimalLongitude	Approximate centre point decimal longitude of the field site in GPS coordinates.
geodeticDatum	The ellipsoid, geodetic datum or spatial reference system (SRS) upon which the geographic coordinates given in decimalLatitude and decimalLongitude are based.
coordinateUncertaintyInMetres	Uncertainty of the coordinates of the centre of the sampling plot.
coordinatePrecision	Precision of the coordinates.
georeferenceSources	A list (concatenated and separated) of maps, gazetteers or other resources used to georeference the Location, described specifically enough to allow anyone in the future to use the same resources.
Table of Species Occurrence	Table with species abundance data (beginning of new table).
id	Unique identification code for species abundance data. Equivalent here to eventID.
type	Type of the record, as defined by the Public Core standard.
licence	Reference to the licence under which the record is published.
institutionID	The identity of the institution publishing the data.
collectionID	The identity of the collection publishing the data.
institutionCode	The code of the institution publishing the data.
collectionCode	The code of the collection where the specimens are conserved.
datasetName	Name of the dataset.
basisOfRecord	The nature of the data record.
recordedBy	A list (concatenated and separated) of names of people, groups or organisations who performed the sampling in the field.
occurrenceID	Identifier of the record, coded as a global unique identifier.
organismQuantity	A number or enumeration value for the quantity of organisms.
organismQuantityType	The type of quantification system used for the quantity of organisms.
sex	The sex and quantity of the individuals captured.
lifeStage	The life stage of the organisms captured.
establishmentMeans	The process of establishment of the species in the location, using a controlled vocabulary: 'naturalised', 'introduced', 'endemic', "unknown".
eventID	Identifier of the events, unique for the dataset.
identifiedBy	A list (concatenated and separated) of names of people, groups or organisations who assigned the Taxon to the subject.
dateIdentified	The date on which the subject was determined as representing the Taxon.
scientificName	Complete scientific name including author and year.
kingdom	Kingdom name.
phylum	Phylum name.
class	Class name.
order	Order name.
family	Family name.
genus	Genus name.
specificEpithet	Specific epithet.
taxonRank	Lowest taxonomic rank of the record.
scientificNameAuthorship	Name of the author of the lowest taxon rank included in the record.

## Additional information

**Results**

We collected a total of 14979 specimens for which 6430 are adults (43%), belonging to 57 species of spiders, 50 genera and 17 families. A total of 14 species are endemic to the Azores Archipelago (9534 specimens; 4145 adults), nine are native non-endemic (3451 specimens; 1538 adults) and 34 are introduced (1976 specimens, 747 adults) (Table 2). Linyphiidae were the richest and most abundant family with 19 (33%) species and 5973 (40%) specimens (Table 2).

**Figure 9.**
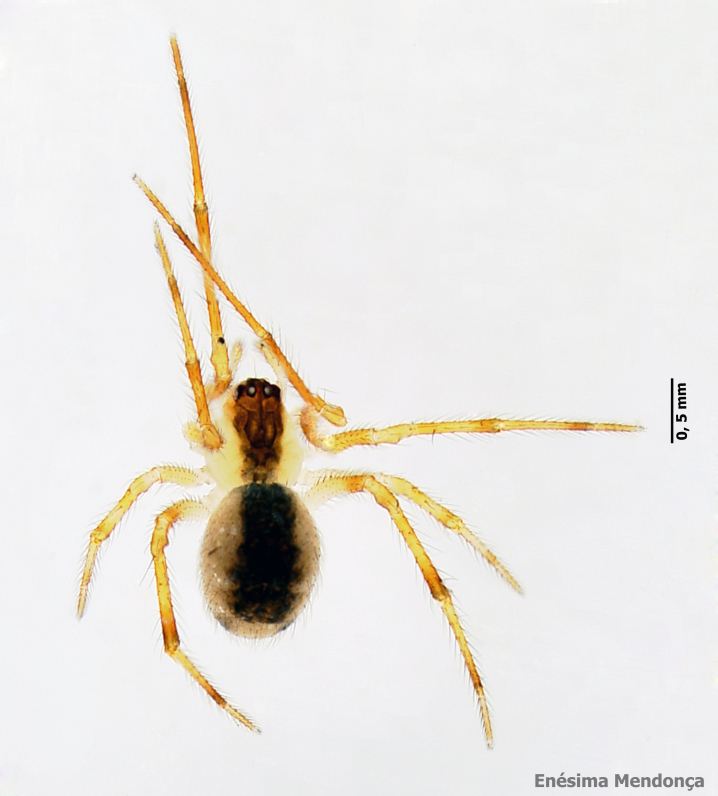
*Rugathodesacoreensis* Wunderlich, 1992 (Credit: Enésima Pereira, Azorean Biodiversity Portal).

**Figure 10.**
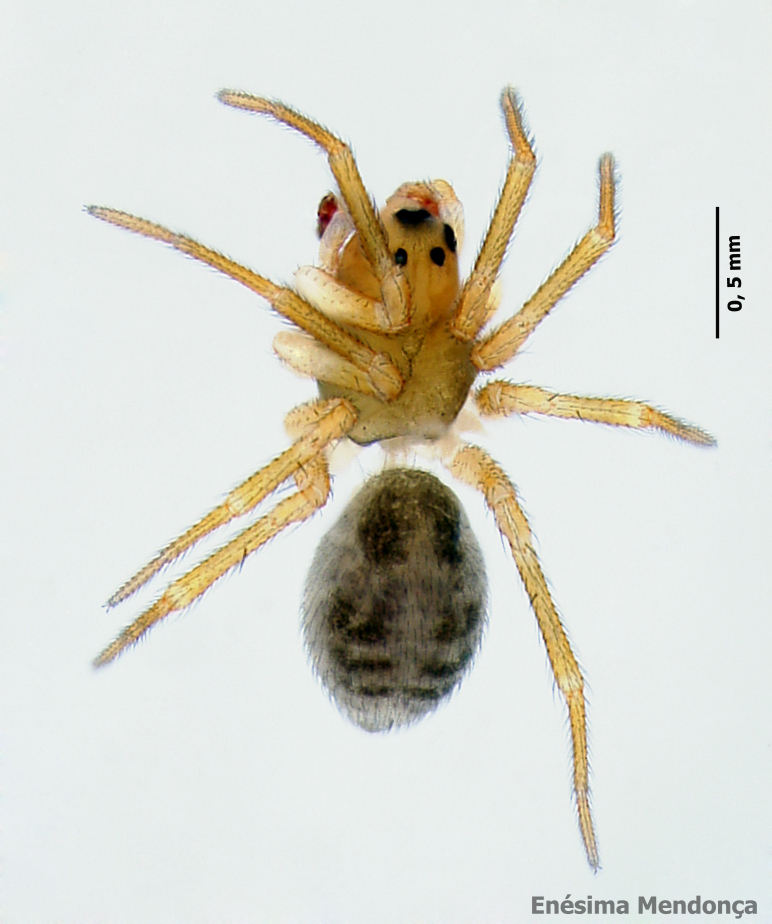
*Savigniorrhipisacoreensis* Wunderlich, 1992 (Credit: Enésima Pereira, Azorean Biodiversity Portal).

The ten most abundant species are composed mostly by endemic or native non-endemic species and only one species is exotic (*Tenuiphantestenuis* (Blackwall, 1852)) (see Table 2). Those ten most abundant species include 84% of all sampled specimens and are clearly the dominant species in the Azorean native forests. The most abundant species were the endemic theridiid *Rugathodesacoreensis* with 3547 specimens (1217 adults) (Fig. 9) and the linyphiid *Savigniorrhipisacoreensis* with 2489 specimens (1003 adults) (Fig. 10).

Four species are new records for the studied Islands: *Textrixcaudata* L. Koch, 1872 was firstly reported from Terceira and Pico Islands, *Araneusangulatus* Clerck, 1757 was firstly reported from Terceira Island, *Nerieneclathrata* (Sundevall, 1830) and *Macaroerisdiligens* (Blackwall, 1867) were firstly reported from Pico Island.

**Figure 11.**
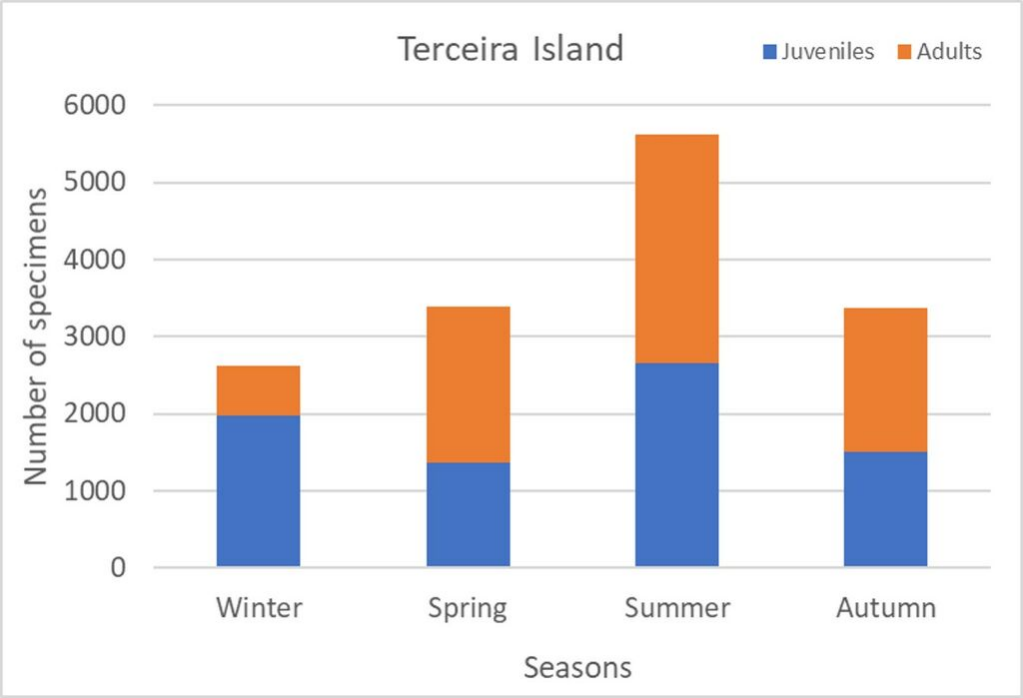
Seasonal abundance of spiders in the studied plots from Terceira Island.

**Figure 12.**
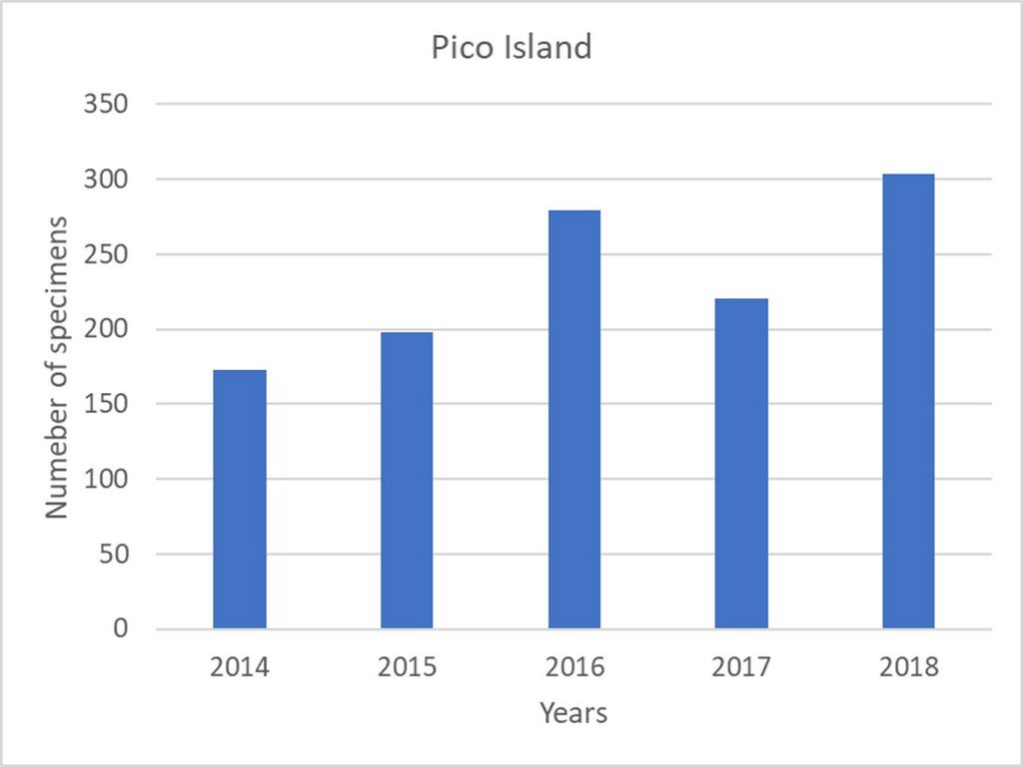
Variation in abundance between years in Pico Island.

**Figure 13.**
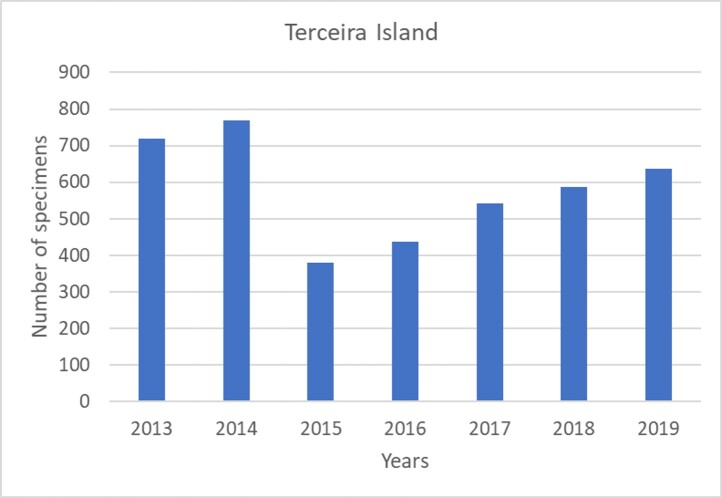
Variation in abundance between years in Terceira Island.

In Terceira Island, the total abundance steadily increases from winter to summer and autumn samples are similar to spring (see Fig. 11). Interestingly, the juveniles dominate in winter (Fig. 11). In Pico Island, there is a tendency to an increase in abundance between 2014 and 2018 (Fig. 12), but in Terceira Island, 2013-2014 were the most productive years (Fig. 13).

**Discussion**

Although SLAM traps were mainly designed to sample flying insects (Borges et al. 2017b), some studies performed in the Azorean Archipelago have used this method to document changes in several arthropod groups over long periods of time. These have focused on understanding patterns of abundance (Borges et al. 2017b, Borges et al. 2020) and beta-diversity (Matthews et al. 2018, Borges et al. 2020), over different seasons and years at the sampled sites. These studies have shown that some of these sites are very well preserved, due to the great prevalence of endemic and native species over exotic (Borges et al. 2017b), with exotic species showing high species temporal turnover (Matthews et al. 2018). However, these still point out that climate change and the spread of exotic species are major threats to the integrity of these areas (Ferreira et al. 2016, Borges et al. 2017b), especially with the increase in diversity of exotic species and a reduction in the abundance of some endemics (Boieiro et al. 2018, Borges et al. 2020).

The data here displayed from Terceira Island were partly used in three publications investigating seasonal patterns (Borges et al. 2017b), patterns in temporal beta diversity for endemic and exotic species (Matthews et al. 2018) and the testing of insect decline trends (Borges et al. 2020). The data from Pico Island are completely novel and are waiting for a more detailed investigation. This kind of data is greatly needed to investigate trends on species diversity and abundance (Borges et al. 2018b) responding to main biodiversity shortfalls (Cardoso et al. 2011) and insect decline patterns (Harvey et al. 2020).

The majority of species collected from Pico and Terceira are common and widespread in the canopies of Azorean endemic trees (Florencio et al. 2013, Florencio et al. 2015, Nunes et al. 2015), which confirms the utility of SLAM traps to monitor canopy-adapted species. However, as the traps were set up fixed in the ground or near the ground, many ground-dwelling species were also sampled, remarkably the two very abundant and widespread *Tenuiphantesmiguelensis* and *T.tenuis*. Particularly relevant is the fact that these two ground-dwelling species are the two more abundant species in Pico Island samples, which clearly differs from Terceira Island patterns, in which specialised canopy species are dominant (see Table 2).

Species diversity, obtained in this monitoring programme (57 species), includes a large fraction of the endemic and native non-endemic species known to occur in Terceira and Pico Islands (Borges et al. 2010), making this data a good representation of the available arachnofauna. Data coming from most of these plots, but sampled with the BALA (Borges et al. 2016) and COBRA (Malumbres-Olarte et al. 2019) protocols, are also available for comparison.

The "SLAM" project here described is creating a unique opportunity not only for theoretical and applied ecology (conservation), but is also contributing to the training of many students in the fields of entomology, ecology and conservation. About 30 students were involved so far in this study (see list above) and were trained in the basics of spider and insect identification and in the quantification of diversity indices. In this way, this project is contributing to solving the taxonomic impediment (Cardoso et al. 2011a). These long-term studies are quite expensive and imply continuous funding from several sources. So far, we have been able to secure around 350 000 Euros since 2012 and until 2024 (see details above). These funds include mostly grants to students and funds to obtain the equipment (SLAM traps and laboratory materials). The cost of the project coordinator (PB) time in fieldwork, student supervision, species identification and project management is here not included.

Hopefully, the continuing funding will allow the monitoring of the unique Azorean native forests for some more years, responding to the need of long-term data to understand the impacts of biodiversity erosion drivers on arthropod diversity in island ecosystems (Borges et al. 2018b, Harvey et al. 2020).

In this way, we are responding to the need for clarifying the impact of invasive species on native species (see, for example, Cardoso et al. 2010), the impact of climate change in Azores (see, for example, Ferreira et al. 2016) and monitoring the status of island native habitats (Borges et al. 2018b). In addition, the data from this project will be important to answer some fundamental questions on island biology, namely those related to community ecology and conservation (see Q29, Q30, Q33, Q39, Q41, Q45 Patiño et al. 2017) and how relative species abundance distributions (SADs) and hyper-dominance are impacted by species diversity at spatial and temporal scales (McGill et al. 2019).

## Figures and Tables

**Table 1.** The list of the twenty sampled sites in the Islands of Pico (n = 7) and Terceira (n = 13).

Island	Habitat	Location ID	Region	Locality	Elevation (m)	Latitude	Longitude
Pico	Mixed Forest	PIC_ML_200	Mistério de St. Luzia	Mistério de St. Luzia Plot 200 m	200	38.53485	-28.43407
Pico	Mixed Forest	PIC_ML_400	Mistério de St. Luzia	Mistério de St. Luzia Plot 400 m	400	38.52075	-28.43112
Pico	Mixed Forest	PIC_ML_600	Mistério de St. Luzia	Mistério de St. Luzia Plot 600 m	600	38.51186	-28.41896
Pico	Mixed Forest	PIC_ML_800	Mistério de St. Luzia	Mistério de St. Luzia Plot 800 m	800	38.49987	-28.42285
Pico	Native Forest	PIC-NFCA-T-09	Pico Caveiro	Caveiro Base	940	38.43775	-28.21065
Pico	Native Forest	PIC-NFLC-T-02	Lagoa do Caiado	Lagoa do Caiado - Euphorbias	816	38.45611	-28.25766
Pico	Native Forest	PIC-NFMP-T-03	Misterio da Prainha	Chão Verde inferior	478	38.48767	-28.27335
Terceira	Erica Forest	TER_0m	Farol da Serreta	Farol da Serreta	46	38.76657	-27.37483
Terceira	Mixed Forest	TER_200m	Mata da Serreta	Serreta 200m	231	38.76037	-27.36380
Terceira	Mixed Forest	TER_400M	Mata da Serreta	Mirador do Pico Carneiro	404	38.76214	-27.34761
Terceira	Native Forest	TER-NFBF-T-01	Biscoito da Ferraria	Labaçal -Morro Assombrado	690	38.76178	-27.21932
Terceira	Native Forest	TER-NFBF-T-02	Biscoito da Ferraria	Chambre A	570	38.75214	-27.23310
Terceira	Native Forest	TER-NFBF-TP41	Biscoito da Ferraria	Pico Alto Nascente	686	38.75019	-27.20725
Terceira	Native Forest	TER-NFPG-T-33	Pico Galhardo	Pico X B	650	38.73341	-27.22711
Terceira	Native Forest	TER-NFSB-T-07	Serra de Santa Bárbara	Lomba	690	38.73725	-27.28994
Terceira	Native Forest	TER-NFSB-T164	Serra de Santa Bárbara	Caldeira - Silvia	890	38.73557	-27.30740
Terceira	Native Forest	TER-NFSB-TE48	Serra de Santa Bárbara	Lagoinha B	744	38.75208	-27.33130
Terceira	Native Forest	TER-NFSB-TE49	Serra de Santa Bárbara	Lagoa Pinheiro B	930	38.74714	-27.31986
Terceira	Native Forest	TER-NFTB-T-15	Terra Brava	Terra Brava -A	630	38.73650	-27.20058
Terceira	Native Forest	TER-NFTB-T-18_Original	Terra Brava	Terra Brava -B	660	38.73206	-27.19720

**Table 2.** The list of studied species mentioning the family, colonisation status (E - endemic from Azores; N - native non-endemic; I - exotic introduced species), IUCN status for the endemic species (VU - Vulnerable; NT - Near Threatened; LC - Least Concern) and indication of an overall abundance (adults + juveniles) in the two studied Islands and total abundance. The new records for a given Island are marked with a (*). The ten most abundant species are in bold.

**Family**	**Species**	**Colonis.**	**Pico**	**Terceira**	**Grand Total**
Agelenidae	*Tegenariadomestica* (Clerck, 1757)	I		1	1
Agelenidae	*Tegenariapagana* C. L. Koch, 1841	I		1	1
Agelenidae	*Textrixcaudata* L. Koch, 1872	I	1 (*)	37 (*)	38
Araneidae	*Agalenatearedii* (Scopoli, 1763)	I		2	2
Araneidae	*Araneusangulatus* Clerck, 1757	I		1 (*)	1
Araneidae	***Gibbaraneaoccidentalis* Wunderlich, 1989**	E (NT)	192	1065	1257
Araneidae	*Mangoraacalypha* (Walckenaer, 1802)	I	1		1
Araneidae	*Zygiellax-notata* (Clerck, 1757)	I	6		6
Cheiracanthiidae	*Cheiracanthiumerraticum* (Walckenaer, 1802)	I	5	32	37
Clubionidae	*Clubionaterrestris* Westring, 1851	I	59	1	60
Clubionidae	*Porrhoclubionadecora* (Blackwall, 1859)	N	77	249	326
Clubionidae	*Porrhoclubionagenevensis* (L. Koch, 1866)	I	9	25	34
Dictynidae	*Emblynaacoreensis* Wunderlich, 1992	E (NT)	2	5	7
Dictynidae	***Lathysdentichelis* (Simon, 1883)**	N	106	896	1002
Dictynidae	*Nigmapuella* (Simon, 1870)	I	4	10	14
Dysderidae	*Dysderacrocata* C.L. Koch, 1838	I	105	197	302
Linyphiidae	***Acorigoneacoreensis* (Wunderlich, 1992)**	E (VU)	354	636	990
Linyphiidae	*Agynetadecora* (O. Pickard-Cambridge, 1871)	I		4	4
Linyphiidae	*Canariphantesacoreensis* (Wunderlich, 1992)	E (VU)	24	24	48
Linyphiidae	*Entelecaraschmitzi* Kulczynski, 1905	I		11	11
Linyphiidae	*Erigoneatra* Blackwall, 1833	I		9	9
Linyphiidae	*Erigoneautumnalis* Emerton, 1882	I		1	1
Linyphiidae	*Erigonedentipalpis* (Wider, 1834)	I		5	5
Linyphiidae	*Mermessusfradeorum* (Berland, 1932)	I	1		1
Linyphiidae	***Microlinyphiajohnsoni* (Blackwall, 1859)**	N	86	456	542
Linyphiidae	*Miniciafloresensis* Wunderlich, 1992	E (VU)	2	13	15
Linyphiidae	*Nerieneclathrata* (Sundevall, 1830)	I	2 (*)		2
Linyphiidae	*Oedothoraxfuscus* (Blackwall, 1834)	I	1	3	4
Linyphiidae	*Palliduphantesschmitzi* (Kulczynski, 1899)	N	23	5	28
Linyphiidae	*Pelecopsisparallela* (Wider, 1834)	I		10	10
Linyphiidae	*Porrhommaborgesi* Wunderlich, 2008	E (VU)		5	5
Linyphiidae	***Savigniorrhipisacoreensis* Wunderlich, 1992**	E (VU)	113	2376	2489
Linyphiidae	***Tenuiphantesmiguelensis* (Wunderlich, 1992)**	N	818	54	872
Linyphiidae	***Tenuiphantestenuis* (Blackwall, 1852)**	I	525	110	635
Linyphiidae	*Walckenaeriagrandis* (Wunderlich, 1992)	E (VU)	39	263	302
Lycosidae	*Arctosaperita* (Latreille, 1799)	I		2	2
Lycosidae	*Pardosaacorensis* Simon, 1883	E (LC)	7	20	27
Mimetidae	*Erofurcata* (Villers, 1789)	I	7	419	426
Pholcidae	*Pholcusphalangioides* (Fuesslin, 1775)	I		3	3
Pisauridae	*Pisauraacoreensis* Wunderlich, 1992	E (NT)	26	101	127
Salticidae	***Macaroeriscata* (Blackwall, 1867)**	N	34	528	562
Salticidae	*Macaroerisdiligens* (Blackwall, 1867)	N	5 (*)	34	39
Salticidae	*Neonacoreensis* Wunderlich, 2008	E (VU)		5	5
Salticidae	*Pseudeuophrysvafra* (Blackwall, 1867)	I		8	8
Salticidae	*Salticusmutabilis* Lucas, 1846	I		6	6
Segestriidae	*Segestriaflorentina* (Rossi, 1790)	I		4	4
Tetragnathidae	*Metellinamerianae* (Scopoli, 1763)	I	5	11	16
Tetragnathidae	***Sancusacoreensis* (Wunderlich, 1992)**	E (VU)	106	594	700
Theridiidae	*Cryptachaeablattea* (Urquhart, 1886)	I	7	204	211
Theridiidae	*Lasaeolaoceanica* Simon, 1883	E (LC)	5	10	15
Theridiidae	*Parasteatodatepidariorum* (C.L. Koch, 1841)	I		8	8
Theridiidae	***Rugathodesacoreensis* Wunderlich, 1992**	E (NT)	210	3337	3547
Theridiidae	*Steatodagrossa* (C.L. Koch, 1838)	I	1	3	4
Theridiidae	*Steatodanobilis* (Thorell, 1875)	I	93	16	109
Theridiidae	*Theridionmusivivum* Schmidt, 1956	N	17	1	18
Thomisidae	*Xysticuscor* Canestrini, 1873	N	30	32	62
Zoropsidae	*Zoropsisspinimana* (Dufour, 1820)	I	18		18
	**Grand Total**		**3126**	**11853**	**14979**

## References

[B7154743] Ávila S. P., Melo C., Berning B., Cordeiro R., Landau B., Silva C. M. (2016). Persististrombus coronatus (Mollusca: Strombidae) in the early Pliocene of Santa Maria Island (Azores: NE Atlantic): palaeoecology, palaeoclimatology and palaeobiogeographic implications on the NE Atlantic Molluscan Biogeographical Provinces. Palaeogeography, Palaeoclimatology, Palaeoecology.

[B7141478] Boieiro Mário, Matthews T. J., Rego Carla, Crespo L. C., Aguiar C. A. S., Cardoso Pedro, Rigal François, Silva Isamberto, Pereira Fernando, Borges P. A. V., Serrano A. R. M. (2018). A comparative analysis of terrestrial arthropod assemblages from a relict forest unveils historical extinctions and colonization differences between two oceanic islands. PLoS One.

[B7139765] Borges P. A. V., Brown V. K. (2001). Phytophagous insects and web-building spiders in relation to pasture vegetation complexity. Ecography.

[B7139792] Borges P. A. V., Brown V. K. (2004). Arthropod community structure in pastures of an island archipelago (Azores): looking for local–regional species richness patterns at fine-scales. Bulletin of Entomological Research.

[B7128649] Borges P. A. V., Aguiar C., Amaral J., Amorim I. R., André G., Arraiol A., Baz A., Dinis F., Enghoff H., Gaspar C., Ilharco F., Mahnert V., Melo C., Pereira F., Quartau J. A., Ribeiro S. P., Ribes J., Serrano A. R. M., Sousa A. B., Strassen R. Z., Vieira L., Vieira V., Vitorino A., Wunderlich J. (2005). Ranking protected areas in the Azores using standardised sampling of soil epigean arthropods. Biodiversity and Conservation.

[B7139881] Borges P. A. V., Lobo J. M., Azevedo E. B., Gaspar C. S., Melo Catarina, Nunes L. V. (2006). Invasibility and species richness of island endemic arthropods: a general model of endemic vs. exotic species. Journal of Biogeography.

[B7139893] Borges P. A. V., Wunderlich Joerg (2008). Spider biodiversity patterns and their conservation in the Azorean archipelago, with descriptions of new species. Systematics and Biodiversity.

[B7139858] Borges P. A. V., Vieira V., Amorim I. R., Bicudo N., Fritzén N., Gaspar C., Heleno R., Hortal J., Lissner J., Logunov D., Machado A., Marcelino J., Meijer S. S., Melo C., Mendonça E. P., Moniz J., Pereira F., Santos A. S., Simões A. M., Torrão E, Borges P. A. V., Costa A., Cunha R., Gabriel R., Gonçalves V., Martins A. M. F., Melo I., Parente M., Raposeiro P., Rodrigues P., Santos R. S., Silva L., Vieira P., Vieira V. (2010). Listagem dos Organismos Terrestres e Marinhos dos Açores. Principia.

[B7152680] Borges P. A. V., Gaspar Clara, Crespo L. C., Rigal François, Cardoso Pedro, Pereira Fernando, Rego Carla, Amorim I. R., Melo Catarina, Aguiar Carlos, André Genage, Mendonça Enésima, Ribeiro Sérvio, Hortal Joaquín, Santos Ana, Barcelos Luís, Enghoff Henrik, Mahnert Volker, Pita Margarida, Ribes Jordi, Baz Arturo, Sousa António, Vieira Virgílio, Wunderlich Jörg, Parmakelis Aristeidis, Whittaker R. J., Quartau J. A., Serrano A. R. M., Triantis K. A. (2016). New records and detailed distribution and abundance of selected arthropod species collected between 1999 and 2011 in Azorean native forests. Biodiversity Data Journal.

[B7152586] Borges P. A. V., Amorim I. R., Terzopoulou S., Rigal F., Emerson B. C., Serrano A. R. M. (2017). Cryptic diversity in the Azorean beetle genus *Tarphius* Erichson, 1845 (Coleoptera: Zopheridae): An integrative taxonomic approach with description of four new species. Zootaxa.

[B7139710] Borges P. A. V., Pimentel R., Carvalho R., Nunes R., Wallon S., Ros Prieto A. (2017). Seasonal dynamics of arthropods in the humid native forests of Terceira Island (Azores). Arquipelago Life and Marine Sciences.

[B7152780] Borges P. A. V., Prez Santa-Rita J. V., Nunes R., Danielczak A., Hochkirch A., Amorim I. R., Lamelas-Lpez L., Karsholt O., Vieira V. (2018). Species conservation profile of moths (Insecta, Lepidoptera) from Azores, Portugal. Biodiversity Data Journal.

[B7139801] Borges P. A. V., Cardoso Pedro, Kreft Holger, Whittaker R. J., Fattorini Simone, Emerson B. C., Gil Artur, Gillespie R. G., Matthews T. J., Santos A. M. C., Steinbauer M. J., Thébaud Christophe, Ah-Peng Claudine, Amorim I. R., Aranda S. C., Arroz A. M., Azevedo J. M. N., Boieiro Mário, Borda-de-Água Luís, Carvalho J. C., Elias R. B., Fernández-Palacios J. M., Florencio Margarita, González-Mancebo J. M., Heaney L. R., Hortal Joaquín, Kueffer Christoph, Lequette Benoit, Martín-Esquivel J. L., López Heriberto, Lamelas-López Lucas, Marcelino José, Nunes Rui, Oromí Pedro, Patiño Jairo, Pérez A. J., Rego Carla, Ribeiro S. P., Rigal François, Rodrigues Pedro, Rominger A. J., Santos-Reis Margarida, Schaefer Hanno, Sérgio Cecília, Serrano A. R. M., Sim-Sim Manuela, Stephenson P. J., Soares A. O., Strasberg Dominique, Vanderporten Alain, Vieira Virgílio, Gabriel Rosalina (2018). Global Island Monitoring Scheme (GIMS): a proposal for the long-term coordinated survey and monitoring of native island forest biota. Biodiversity and Conservation.

[B7139741] Borges P. A. V., Rigal François, Ros‐Prieto Alejandra, Cardoso Pedro (2020). Increase of insular exotic arthropod diversity is a fundamental dimension of the current biodiversity crisis. Insect Conservation and Diversity.

[B7139301] Borges P. A. V., Costa R. (2021). Long-term monitoring of Azorean forest spiders. v.1.4. Universidade dos Açores. Dataset/Samplingevent.

[B7151249] Cardoso Pedro, Aranda S. C., Lobo J. M., Dinis Francisco, Gaspar Clara, Borges P. A. V. (2009). A spatial scale assessment of habitat effects on arthropod communities of an oceanic island. Acta Oecologica.

[B7139902] Cardoso Pedro, Arnedo M. A., Triantis K. A., Borges P. A. V. (2010). Drivers of diversity in Macaronesian spiders and the role of species extinctions. Journal of Biogeography.

[B7154365] Cardoso Pedro, Erwin T. L., Borges P. A. V., New T. R. (2011). The seven impediments in invertebrate conservation and how to overcome them. Biological Conservation.

[B7156937] Cardoso Pedro, Borges P. A. V., Triantis K. A., Ferrández M. A., Martín J. L. (2011). Adapting the IUCN Red List criteria for invertebrates. Biological Conservation.

[B7312682] Cicconardi Francesco, Borges P. A. V., Strasberg Dominique, Oromí Pedro, López Heriberto, Pérez-Delgado A. J., Casquet Juliane, Caujapé-Castells Juli, Fernández-Palacios J. M., Thébaud Christophe, Emerson B. C. (2017). MtDNA metagenomics reveals large-scale invasion of belowground arthropod communities by introduced species. Molecular Ecology.

[B7139920] Crespo L. C., Bosmans R., Cardoso P., Borges P. A. V. (2013). On the endemic spider species of the genus *Savigniorrhipis* Wunderlich, 1992 (Araneae: Linyphiidae) in the Azores (Portugal), with description of a new species.. Zootaxa.

[B7139929] Crespo L. C., Bosmans R., Cardoso P., Borges P. A. V. (2014). On three endemic species of the linyphiid spider genus *Canariphantes* Wunderlich, 1992 (Araneae, Linyphiidae) from the Azores archipelago. Zootaxa.

[B7312672] Crespo L. C., Enguidanos A., Silva I., Cardoso P. ., Arnedo M. A. (2021). The Atlantic connection: coastal habitat favoured long distance dispersal and colonization of Azores and Madeira by Dysdera spiders (Araneae: Dysderidae). Systematics and Biodiversity.

[B7154754] Elias R. B., Gil A., Silva L., Fernndez-Palacios J. M., Azevedo E. B., Reis F. (2016). Natural zonal vegetation of the Azores Islands: characterization and potential distribution. Phytocoenologia.

[B7150563] Ferreira M. T., Cardoso Pedro, Borges P. A. V., Gabriel Rosalina, de Azevedo E. B., Reis Francisco, Araújo M. B., Elias R. B. (2016). Effects of climate change on the distribution of indigenous species in oceanic islands (Azores). Climatic Change.

[B7139948] Florencio M., Cardoso Pedro, Lobo J. M., de Azevedo E. B., Borges P. A. V. (2013). Arthropod assemblage homogenization in oceanic islands: the role of indigenous and exotic species under landscape disturbance.. Diversity and Distributions.

[B7151228] Florencio M., Lobo J. M., Cardoso P., Almeida-Neto M., Borges P. A. V. (2015). The Colonisation of Exotic Species Does Not Have to Trigger Faunal Homogenisation: Lessons from the Assembly Patterns of Arthropods on Oceanic Islands. PLoS One.

[B7153271] Gaspar Clara, Gaston K. J., Borges P. A. V., Cardoso Pedro (2010). Selection of priority areas for arthropod conservation in the Azores archipelago. Journal of Insect Conservation.

[B7140005] Gaspar Clara, Gaston K. J., Borges P. A. V., Cardoso Pedro (2011). Selection of priority areas for arthropod conservation in the Azores archipelago. Journal of Insect Conservation.

[B7152653] Gaston K. J., Borges P. A. V., He F., Gaspar C. (2006). Abundance, spatial variance and occupancy: arthropod species distribution in the Azores. Journal of Animal Ecology.

[B7154375] Harvey J. A., Heinen Robin, Armbrecht Inge, Basset Yves, Baxter-Gilbert J. H., Bezemer T. M., Böhm Monika, Bommarco Riccardo, Borges P. A. V., Cardoso Pedro, Clausnitzer Viola, Cornelisse Tara, Crone Elizabeth E., Dicke Marcel, Dijkstra K. -D. B., Dyer Lee, Ellers Jacintha, Fartmann Thomas, Forister M. L., Furlong Mi. J., Garcia-Aguayo Andres, Gerlach Justin, Gols Rieta, Goulson Dave, Habel Jan-Christian, Haddad N. M., Hallmann C. A., Henriques Sérgio, Herberstein M. E., Hochkirch Axel, Hughes A. C., Jepsen Sarina, Jones T. H., Kaydan B. M., Kleijn David, Klein Alexandra-Maria, Latty Tanya, Leather S. R., Lewis S. M., Lister B. C., Losey J. E., Lowe E. C., Macadam C. R., Montoya-Lerma James, Nagano C. D., Ogan Sophie, Orr Michael C., Painting C. J., Pham Thai-Hong, Potts S. G., Rauf Aunu, Roslin T. L., Samways M. J., Sanchez-Bayo Francisco, Sar Sim A., Schultz C. B., Soares A. O., Thancharoen Anchana, Tscharntke Teja, Tylianakis J. M., Umbers K. D. L., Vet L. E. M., Visser M. E., Vujic Ante, Wagner D. L., WallisDeVries M. F., Westphal Catrin, White T. E., Wilkins V. L., Williams P. H., Wyckhuys K. A. G., Zhu Zeng-Rong, de Kroon Hans (2020). International scientists formulate a roadmap for insect conservation and recovery. Nature Ecology & Evolution.

[B7157592] Macías-Hernández Nuria, Ramos Cândida, Domènech Marc, Febles Sara, Santos Irene, Arnedo Miquel, Borges P. A. V., Emerson B. C., Cardoso Pedro (2020). A database of functional traits for spiders from native forests of the Iberian Peninsula and Macaronesia. Biodiversity Data Journal.

[B7140014] Magurran A. E., Baillie S. R., Buckland S. T., Dick J. McP., Elston D. A., Scott E. M., Smith R. I., Somerfield P. J., Watt A. D. (2010). Long-term datasets in biodiversity research and monitoring: assessing change in ecological communities through time. Trends in Ecology & Evolution.

[B7140045] Malumbres-Olarte Jagoba, Cardoso Pedro, Crespo L. C., Gabriel Rosalina, Pereira Fernando, Carvalho Rui, Rego Carla, Nunes Rui, Ferreira Maria, Amorim Isabel, Rigal François, Borges P. A. V. (2019). Standardised inventories of spiders (Arachnida, Araneae) of Macaronesia I: The native forests of the Azores (Pico and Terceira islands). Biodiversity Data Journal.

[B7140070] Marcelino José, Borges Paulo, Borges Isabel, Pereira Enésima, Santos Vasco, Soares António (2021). Standardised arthropod (Arthropoda) inventory across natural and anthropogenic impacted habitats in the Azores archipelago. Biodiversity Data Journal.

[B7139731] Matthews Thomas J., Sadler Jon, Carvalho Rui, Nunes Rui, Borges P. A. V. (2018). Differential temporal beta‐diversity patterns of native and non‐native arthropod species in a fragmented native forest landscape. Ecography.

[B7152722] Matthews T. J., Borregaard M. K., Ugland K. I., Borges P. A. V., Rigal François, Cardoso Pedro, Whittaker R. J. (2014). The gambin model provides a superior fit to species abundance distributions with a single free parameter: evidence, implementation and interpretation. Ecography.

[B7156981] McGill B. J., Chase J. M., Hortal Joaquín, Overcast Isaac, Rominger A. J., Rosindell James, Borges P. A. V., Emerson B. C., Etienne R. S., Hickerson M. J., Mahler D. L., Massol Francois, McGaughran Angela, Neves Pedro, Parent Christine, Patiño Jairo, Ruffley Megan, Wagner C. E., Gillespie Rosemary (2019). Unifying macroecology and macroevolution to answer fundamental questions about biodiversity. Global Ecology and Biogeography.

[B7140081] Nogué Sandra, de Nascimento Lea, Froyd C. A., Wilmshurst J. M., de Boer E. J., Coffey Emily E. D., Whittaker R. J., Fernández-Palacios J. M., Willis K. J. (2017). Island biodiversity conservation needs palaeoecology. Nature Ecology & Evolution.

[B7152967] Nunes R., Gabriel R., Elias R. B., Rigal F., Soares A. O., Cardoso P., Borges P. A. V. (2015). Arthropods and other Biota associated with Azorean Trees & Shrubs: Juniperusbrevifolia. Arquipelago Life and Marine Sciences.

[B7140116] Parmakelis Aristeidis, Rigal François, Mourikis Thanos, Balanika Katerina, Terzopoulou Sofia, Rego Carla, Amorim I. R., Crespo Luís, Pereira Fernando, Triantis K. A., Whittaker R. J., Borges P. A. V. (2015). Comparative phylogeography of endemic Azorean arthropods. BMC Evolutionary Biology.

[B7156947] Patiño Jairo, Whittaker R. J., Borges P. A. V., Fernández-Palacios J. M., Ah-Peng Claudine, Araújo M. B., Ávila S. P., Cardoso Pedro, Cornuault Josselin, de Boer E. J., de Nascimento Lea, Gil Artur, González-Castro Aarón, Gruner D. S., Heleno Ruben, Hortal Joaquín, Illera J. C., Kaiser-Bunbury C. N., Matthews T. J., Papadopoulou Anna, Pettorelli Nathalie, Price J. P., Santos A. M. C., Steinbauer M. J., Triantis K. A., Valente Luis, Vargas Pablo, Weigelt Patrick, Emerson B. C. (2017). A roadmap for island biology: 50 fundamental questions after 50 years of The Theory of Island Biogeography. Journal of Biogeography.

[B7140133] Pearce J. L., Venier L. A. (2006). The use of ground beetles (Coleoptera: Carabidae) and spiders (Araneae) as bioindicators of sustainable forest management: A review. Ecological Indicators.

[B7152952] Ribeiro S. P., Borges P. A. V., Gaspar Clara, Melo Catarina, Serrano A. R. M., Amaral João, Aguiar Carlos, André Genage, Quartau J. A. (2005). Canopy insect herbivores in the Azorean Laurisilva forests: key host plant species in a highly generalist insect community. Ecography.

[B7152662] Rigal François, Whittaker R. J., Triantis K. A., Borges P. A. V. (2013). Integration of non-indigenous species within the interspecific abundance–occupancy relationship. Acta Oecologica.

[B7151523] Rigal François, Cardoso Pedro, Lobo J. M., Triantis K. A., Whittaker R. J., Amorim I. R., Borges P. A. V. (2017). Functional traits of indigenous and exotic ground-dwelling arthropods show contrasting responses to land-use change in an oceanic island, Terceira, Azores. Diversity and Distributions.

[B7152863] World-Spider-Catalog (2021). Natural History Museum Bern, online at.

